# Growth Factors Released from Advanced Platelet-Rich Fibrin in the Presence of Calcium-Based Silicate Materials and Their Impact on the Viability and Migration of Stem Cells of Apical Papilla

**DOI:** 10.3390/dj11090220

**Published:** 2023-09-19

**Authors:** Cristine Smoczer, Kenneth R. Yuth, Mazin A. Askar, Laura A. Young, Susan B. Paurazas

**Affiliations:** 1 Division of Integrated Biomedical Sciences, University of Detroit Mercy School of Dentistry, Detroit, MI 48208, USA; smoczecr@udmercy.edu (C.S.);; 2 Graduate Endodontics, University of Detroit Mercy School of Dentistry, Detroit, MI 48208, USAaskarma@udmercy.edu (M.A.A.)

**Keywords:** advanced platelet-rich fibrin, stem cells of the apical papilla, growth factors, calcium-based silicate materials, regenerative endodontic procedures

## Abstract

Advanced platelet-rich fibrin (A-PRF) provides the scaffold and growth factors necessary for stem cells to proliferate and differentiate in successful regenerative endodontic procedures. This study investigates the release of transforming growth factor-β1 (TGF-β1), platelet-derived growth factor (PDGF), and vascular endothelial growth factor (VEGF) from A-PRF in cell culture media in the presence and absence of mineral trioxide aggregate (MTA) or Biodentine. Additionally, this research assesses the viability and migration of stem cells of the apical papilla (SCAP) in previously conditioned media. A-PRF obtained from 14 participants were incubated for 7 days in cell culture media alone or via layering with MTA or Biodentine discs and the release of selected growth factors in the media was evaluated using ELISA. The viability of SCAP grown in conditioned media was measured using the CCK8 assay, while SCAP migration was assessed via a transwell assay by counting migrated cells. The release of TGF-β1, PDGF, and VEGF was significantly higher in media with A-PRF alone than in the presence of either calcium-based silicate material (*p* < 0.05), which showed no difference from the no-A-PRF control (*p* < 0.05). None of the tested growth factors released in the A-PRF-conditioned media correlated with clot weight. A-PRF-conditioned media, both with and without calcium-based silicate materials, did not impact SCAP viability and migration (*p* > 0.05). This study shows that SCAP behavior is not impacted by the decrease in growth factor released in the presence of calcium-based silicate materials and that their role in REPs warrants further investigation.

## 1. Introduction

Immature permanent teeth with necrotic pulp often present difficulties for endodontic treatment due to thin and weak dentinal walls and the subsequent risk of stress-overload fracture [1,2]. In addition, they may have open apices, which present significant challenges in terms of cleaning and obturation using traditional methods [3]. In recent years, the regeneration of damaged pulp, which promotes the remineralization and thickening of the dentinal walls in order to strengthen tooth structures, has been attempted via regenerative endodontic procedures (REPs) [4,5]. REPs apply the concept of tissue bioengineering, relying on the triad of stem cells, scaffolds, and growth factors synergistically interacting in the canal space, and promote pulp–dentin regeneration [6,7,8]. Numerous studies demonstrate the induction of continued root development with high survival and healing rates through regenerative endodontic procedures (REPs) [9,10,11]. The scaffold and growth factors necessary for stem cell growth and differentiation can be provided by blood clots produced via invoked bleeding into the periapical space caused by over-instrumentation [12]. Failure to achieve apical bleeding or produce an adequate blood volume remains a common challenge in REPs [13,14]. An alternative to provoked bleeding is the use of platelet concentrates that, although more difficult to produce, are enriched in blood cells, fibrin, growth factors, pro-inflammatory cytokines, and anti-microbial peptides [15]. A limitation that applies to all platelet-rich concentrate therapies is the potential difficulty of drawing venous blood, especially in the pediatric patients that provide the majority of cases approached through REPs [16].

Various methods have been developed to produce platelet concentrates over the years. The first-generation concentrate was platelet-rich plasma (PRP). This was shown to enhance wound healing, but required the addition of anti-coagulants during blood collection [16]. The second generation of platelet concentrate relied on a lower centrifugation speed and a higher centrifugation time of blood collected without artificial additives [17,18]. This method produces advanced platelet-rich fibrin (A-PRF), a porous structure with an even distribution of platelets and leukocytes throughout [19,20]. The large number of platelets leads to an increased and constant release of growth factors over time, such as transforming growth factor beta (TGF-β1), platelet-derived growth factor (PDGF), and vascular endothelial growth factor (VEGF) [19,21]. Growth factors are necessary to induce a homing in on resident stem cells, as well as their proliferation and differentiation [7].

The stem cells used in REPs are dental pulp stem cells (DPSCs) or stem cells from the apical papilla (SCAP). SCAP are mesenchymal stem cells that reside at the apex of the incompletely developed root. They have faster doubling time and mineralization capacity than DPSCs, indicating superior potency and differentiation potential [22,23,24]. Given these properties and ease of access during REPs, the apical papilla could be the best stem cell source for pulp tissue engineering [22,25,26].

During REPs, the root canal is emptied of necrotic tissue and, disinfected, is filled by the provoked blood clot or a platelet concentrate scaffold, with calcium-based silicate materials providing an artificial barrier to create a microenvironment for improved periapical healing [11]. Mineral trioxide aggregate (MTA) and Biodentine™ (BD) are calcium-based silicate materials that have recently been used in conjunction with PRF and A-PRF in REPs to due to their biocompatibility, ease of handling, and capability to stimulate tissue healing without inflammation [11,27,28,29,30]. Both materials were shown to have antibacterial activity, and the subsequent biofilm reduction improved TGF-β1 release from PRF [31,32,33]. Using PRF and calcium silicate-based materials in REPs in vivo caused a significant improvement of symptoms, thickening of dentinal walls, progression of root formation, and apical closure with the resolution of apical periodontitis [11,27].

Therefore, it is imperative to understand the combined effects of calcium silicate-based materials, SCAP, and A-PRF with regard to the objective of regenerating dentin-like tissue and restoring normal tooth function [34]. Previous studies investigated the effect of calcium silicate-based materials on SCAP proliferation [11,35] and their effect on the growth factors released from PRF [36]; however, the way in which calcium silicate-based materials affect the release of growth factors and influence SCAP behavior remains to be evaluated.

The aim of this study is to compare release of VEGF, PDGF, and TGF-B1 from A-PRF when combined with MTA or BD, and the effect of these factors on SCAP viability and migration.

## 2. Materials and Methods

### 2.1. Subjects and Sample Collection

This study was reviewed and approved by the University of Detroit Mercy ethical review board (IRB-C-#21-22-51). Subjects were recruited from dental students at the University of Detroit Mercy School of Dentistry who volunteered their participation. Prior to sample collection, all participants were informed of the purpose of the study and asked to sign an informed consent form. Volunteers eligible for inclusion were healthy males, ages 20–40 years old, who were not taking any medication or supplements.

Fourteen volunteers meeting the inclusion criteria were enrolled in the study. Three test tubes of 10 mL peripheral blood were collected from the antecubital vein of each participant by a registered nurse. Blood collection was carried out on two separate occasions with seven subjects included in each round.

### 2.2. A-PRF Preparation and Media Conditioning

All blood samples collected in Solid-PRF tubes with no additives (Bio-PRF) were immediately centrifuged at 1400 rpm (208× *g*) for 14 min [17,19] in a horizontal PRF centrifuge machine (Bio-PRF, Venice, FL, USA). Following layer separation, A-PRF clots were excised, and excess serum was compressed using a sterile gauze [36]. The three clots from the same participant were weighted and trimmed to ensure the same weight A-PRF for each experimental group.

The three A-PRF clots obtained from one donor were each placed in a sterile 10 mL tube as follows: A-PRF clot alone, A-PRF clot + BD disc, and A-PRF clot + MTA disc and 5 mL of complete cell culture media were added to each tube. Cell culture media alone served as the control after treatment in the same way as other groups. All samples were incubated for 7 days at 37 °C and 5% CO_2_ and conditioned media were filtered using a 0.22 μm-pore-size syringe filter (Millipore, Burlington, MA, USA) prior to use.

### 2.3. Growth Factor Quantification

The release of three growth factors (GFs) in the 7-day-conditioned media from all four groups was evaluated using enzyme-linked immunosorbent assay (ELISA) kits specific to each GF (R&D Systems, Minneapolis, MN, USA). PDGF-AB (DHD00C), TGF-β1 (DB100C), and VEGF (DVE00) were measured according to the manufacturer’s recommendations.

### 2.4. Cell Culture and Reagent Preparation

Characterized stem cells of the apical papilla (SCAP) (RP-89) [37] were cultured in α-MEM (Gibco, Grand Island, NY, USA) supplemented with 10% fetal bovine serum (Gemini, West Sacramento, CA, USA) and 1% penicillin/ streptomycin (Gemini). Cells were grown at 37 °C and 5% CO_2_ and used in subsequent experiments at passages 5–8.

MTA (ProRoot MTA, Dentsply Sirona, Charlotte, NC, USA) and BD (Septodont, Lancaster, PA, USA) were mixed according to the manufacturer’s recommendations and placed in pre-sterilized Teflon molds (with 5 mm diameter and a thickness of 3 mm) to produce 14 MTA and 14 BD discs. Discs hardened when covered with moist gauze and were UV-sterilized for 4 h prior to use for media conditioning.

### 2.5. Cell Viability Assay

The effect of PRF on cell viability, both with and without calcium silicate-based materials, was quantitatively evaluated using the cell counting kit-8 (CCK-8) (Abcam, Waltham, MA, USA) as per the manufacturer’s protocol. SCAP were seeded in a clear-bottom 96-well plate at a density of 5000 cells/well and incubated for 48 h in 100 μL of conditioned media collected from the 4 groups. After incubation, CCK-8 solution was added to each well and absorbance was measured at 450 nm using a Tecan Spark plate reader.

### 2.6. Cell Migration Assay

To evaluate SCAP migration in conditioned media, 2 × 10^4^ cells/well were plated in the inserts of the Corning™ Transwell™24-Well Plate with 8.0 μm-pore permeable polycarbonate membrane (Corning, Corning, NY, USA) in plain α-MEM. Conditioned media from the 4 groups were added to the lower chambers and cells were allowed to migrate for 24 h. Migrated cells that moved to the lower surface of the membrane were fixed with ethanol and stained with 1% crystal violet. Images of five random fields of view for each membrane were captured via light microscopy. The ImageJ software (National Institutes of Health, Stapleton, NY, USA) [38] was used for the initial count of the crystal violet-stained migrated cells in those photographs. Additionally, those counts were manually verified by a researcher that had been blinded to the groups.

### 2.7. Statistical Analysis

Power analysis was conducted to determine the appropriate sample size prior to data acquisition. Parameter estimates for the sample size analyses were derived using growth factor release and migration data from Hong et al. [39]. It was estimated that at least 14 observations per treatment group were needed (i.e., total N =56) in order to achieve 80% power for the overall ANOVA test with alpha = 0.05.

Data analysis was performed using the GraphPad Prism 9.0 software package, with data expressed as the mean value ± standard error and the significance level set to *p* < 0.05. One-way analysis of variance (ANOVA) and post hoc Tukey’s multiple comparison tests were performed to compare the means between the four groups. A linear regression analysis was followed by a Spearman correlation analysis to determine the strength of the correlation between clot weight and each growth factor.

## 3. Results

### 3.1. Release of Growth Factors

**Figure 1:**
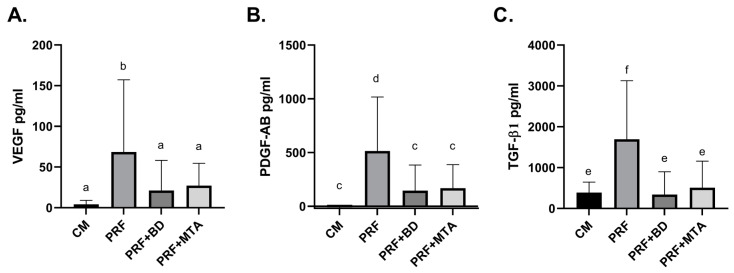
Quantification of growth factors. The release of growth factors from A-PRF incubated for 7 days in cell culture medium alone or in the presence of MTA or BD was evaluated by growth factor specific ELISA kits for (**A**) VEGF (a: *p* > 0.05, b: *p* < 0.05) (**B**) PDGF-AB (c: *p* > 0.05, d: *p* < 0.001), and (**C**) TGF-β1 (e: *p* > 0.05, f: *p* < 0.001). Absolute growth factor levels were measured in duplicate and represented as means of the 14 samples +/− standard deviation. One-way ANOVA and post hoc Tukey’s multiple comparison test were performed. CM (control medium); PRF (A-PRF-conditioned medium); PRF + BD (A-PRF- and Biodentine-conditioned medium); PRF + MTA (A-PRF- and MTA-conditioned medium).

The release of three growth factors: VEGF, PDGF-AB, and TGF-β1 in cell culture media conditioned for seven days using A-PRF with and without the calcium silicate-based materials was assessed via specific ELISA assays. The amount of VEGF released in the A-PRF alone group was significantly higher than the amount released in the presence of either MTA or BD (b, *p* < 0.05). However, there was no difference between the BD and MTA groups, which were also no different from control (a, *p* > 0.05) (Figure 1A). PDGF-AB levels were highest in the A-PRF group and significantly higher than those in the calcium silicate-based material groups (d, *p* < 0.001), which were no different from each other or the control (c, *p* > 0.05) (Figure 1B). A-PRF alone also released the highest amounts of TGF-β1 (f, *p* < 0.001). The groups of A-PRF with the different calcium silicate-based materials were not different from each other or the control in terms of the amount of TGF-β1 released in the media (e, *p* > 0.05) (Figure 1C).

### 3.2. Correlation of Growth Factors with A-PRF Weight

**Figure 2:**
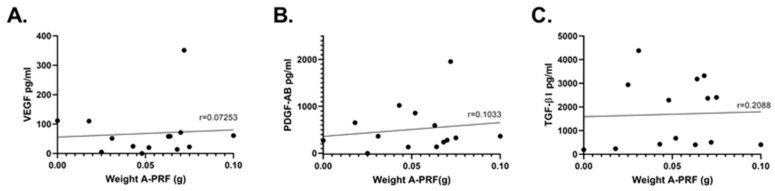
Correlation between A-PRF weight and growth factor levels in the A-PRF-only group. A linear regression analysis was performed to correlate the A-PRF weight for each of the 14 samples with the respective release of growth factors assessed by ELISA: VEGF (**A**); PDGF (**B**); and TGF-β1 (**C**). A Spearman correlation analysis was used to determine the strength of the correlation for each growth factor. r = correlation coefficient.

The size of expressions of A-PRF was highly variable among participants, ranging from very small (0.01 g) to very large (0.1 g) clots. Although the amounts of VEGF, PDGF-AB, and TGF-β1 released from A-PRF in media over 7 days also displayed large variability, there was no correlation between the weight of the blood clots and the amount of factor released in media (Figure 2A–C). Spearman analysis of correlation showed no statistically significant correlation of growth factor and size of A-PRF.

### 3.3. SCAP Viability and Migration

**Figure 3:**
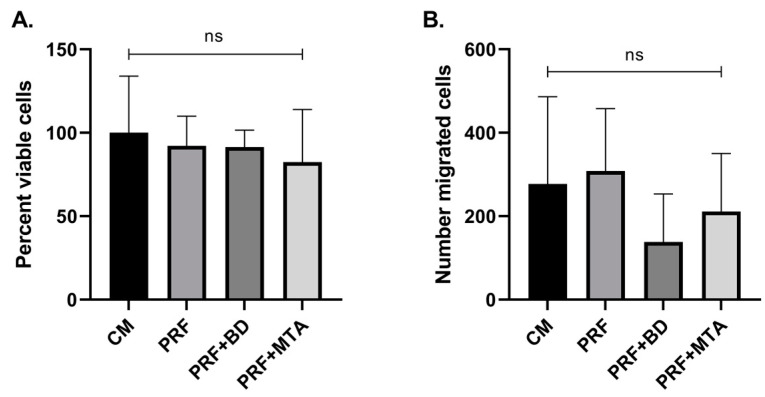
SCAP viability and migration analysis. The impact of conditioned media with A-PRF in the presence or absence of calcium silicate-based materials on SCAP was evaluated via specific assays. (**A**) Viability of SCAP after 48 h growth in conditioned media was assessed via CCK-8. Absorbance was measured at 450 nm and results were reported as the percent of viable cells when the control group was set to 100%. (**B**) Migration of SCAP was analyzed using a Transwell assay where cells that migrated toward conditioned media after 24 h were stained with crystal violet and counted in five fields of view per sample. All data were reported as mean of the 14 samples +/− standard deviation and statistical significance among groups was determined using one-way ANOVA with post hoc Tukey’s multiple comparison tests. CM (control medium); PRF (A-PRF-conditioned medium); PRF + BD (A-PRF and BD-conditioned medium); PRF + MTA (A-PRF and MTA-conditioned medium). ns, not significant (*p* > 0.05).

To determine the effect of the growth factors released in media with A-PRF in the presence and absence of MTA and BD, we evaluated the viability of SCAP grown in conditioned media for 48 h using the CCK-8 assay. Cell viability in all groups was approximately 100%, with no significant difference seen among the groups (not significant (ns) *p* > 0.05) (Figure 3A).

SCAP migratory potential in conditioned media from the four groups was evaluated using a Transwell migration assay. Despite the difference in growth factor release among the groups, there was no significant difference in the migration pattern, although a trend toward a marginally lower number of migratory cells was observed in the BD-conditioned media (not significant (ns) *p* > 0.05) (Figure 3B).

## 4. Discussion

PRF and its derivative, A-PRF, have been used widely in regenerative medicine and dentistry to provide organic autologous scaffolds and to release the high concentrations of growth factors necessary for tissue regeneration [40]. In this study, we evaluated the concentration of three growth factors (VEGF, PDGF-AB, and TGF-β1) released from A-PRF in cell culture media in the presence and absence of calcium silicate-based materials over 7 days. We determined that the levels of all three growth factors were significantly higher in the absence of either MTA or BD, which had levels slightly above those of the negative control. In the second part of our study, we examined the viability and migration of SCAP grown in media conditioned with A-PRF both with and without calcium silicate-based materials. Interestingly, there were no significant differences among groups for both parameters.

Since A-PRF size is highly variable among individuals, correlating with age and possibly gender [41], we had strict inclusion criteria and conducted a power analysis prior to conducting the experiment to ensure the study was well-powered. We determined that the appropriate sample size for detecting statistical significance was 14 participants. As expected, we found A-PRF weights obtained from individual volunteers to be highly variable; however, we found no correlation of the A-PRF weight with the amount of growth factors released in media over seven days. This could be due to the variability in the number of platelets and leukocytes trapped in the fibrin mesh since growth factor release correlates with platelet counts [41].

Calcium silicate-based materials such as MTA and BD are used in multiple facets of dentistry due to their biocompatibility, conductive and inductive properties, and desirable handling characteristics [29]. MTA sets in at about 4–5 h, while BD (an MTA-like agent) has similar mechanical properties and biocompatibility with a shorter setting time (6 min) and easier handling. Here, we focused the first part of our study on the impact of these biomaterials on the release of growth factor from A-PRF. Previous studies showed that PDGF-AB secretion peaks at day 1, TGF-β1 at day 7, and VEGF around day 3 [21,42]. Because of these differences in the release kinetics for the selected growth factors, we decided to conduct a single reading of the cumulative amounts at 7 days, following the TGF-β1 peak. With this approach, we ensured that the peak release was included for all growth factors. The levels of VEGF, TGF-β1, and PDGF-AB were drastically reduced in media with A-PRF and either MTA or BD when compared to media with A-PRF alone. This contrasts the results of a study reporting an increase in the release of TGF-β1 when layered with BD and PRF, although the incubation period was limited to 5 h [36]. The decreased levels of VEGF were also in contrast to a previous study, which showed a significant increase in VEGF concentrations after 3 days of exposure to MTA and BD [43]. Our choice to evaluate the cumulative release of growth factors at 7 days could account for some differences in outcomes with other publications. We suspected that conditioning media for shorter time periods would have introduced more variability into the results since not all growth factors would have reached their peak release point [21,42]. Longer time periods would have probably produced similar results as the release plateaus or decreases after the 7 days.

Since the pH values of BD and MTA are 12 and 10.1, respectively [31,44], a longer exposure to an alkaline environment, such as in our study, could have induced potential degradation and a subsequent decrease in the detection of the growth factors. Conformational changes in the protein structure of growth factors could alter the binding ability to antibodies in ELISA, which could explain the observed decrease in the calcium silicate-based material-conditioned media. Zirconium oxide, used as a radioopacifier in BD, is a bioinert material. This is in contrast to bismuth oxide, the radioopacifier in MTA. Previous studies have shown the long-term release of bismuth oxide into the neighboring tissues and its interaction with other substances, which could potentially explain its influence on the release of growth factors in conditioned media [45,46]. A time course of growth factor release during a seven-day period could probably enable a better understanding of the effects of long-term exposure to an alkaline environment.

We also examined the viability and migration of SCAP grown in media conditioned for 7 days with A-PRF with and without calcium silicate-based materials and found that conditioned media had no impact on either parameter. PRF was shown to have a cell-specific effect on cell proliferation and not to induce proliferation in human mesenchymal stem cells, such as SCAP [47,48]. A higher platelet concentration has been shown to inhibit proliferation and migration, suggesting a compromise is necessary between high and low platelet concentrations to obtain an optimal healing setting [15,49]. Since platelet counts are correlated with growth factors, this may imply an optimal range of growth factor concentration that promotes favorable outcomes at the cellular level [41]. Also, MTA and BD have been reported to have variable effects on SCAP proliferation and migration. These are highly dependent on the method of assessment and whether they are used in direct contact with the cells or to condition cell growth media [50]. Similar to our findings, previous studies have shown no difference in cell proliferation and differentiation potential when exposed to either bismuth or zirconium oxide, the radioopacifiers present in MTA and BD, respectively [51,52]. We investigated the effect of media conditioned using A-PRF with and without calcium silicate-based materials on the viability and migration of SCAP, thus offering a broader view of the combined interactions of the tissue engineering components of REPs. Now that their role providing a barrier against microorganisms with little cytotoxicity has been established [53], future studies should further investigate the role of calcium silicate-based materials in REPs by evaluating their impact on SCAP proliferation and odonto/osteoblastic differentiation.

## 5. Conclusions

This study showed that SCAP are not impacted by the decrease in growth factor release from A-PRF in the presence of MTA or BD. Within its limitations, this in vitro study supports the addition of calcium silicate-based materials in the model of the tissue engineering triad in terms of regenerative endodontic procedures.

## Data Availability

Data is unavailable due to privacy.
